# Supplementary material for: Recombinant protein platform for high-throughput investigation of peptide-liposome interactions via fluorescence anisotropy depolarization
Source: Commun Chem. 2026 Apr 2;9:165. doi: 10.1038/s42004-026-01994-9 (PMC13106830; doi:10.1038/s42004-026-01994-9)
Supplement: Supplementary file 2 — Supplementary information for Recombinant protein platform for high-throughput investigation of peptide-liposome interactions via fluorescence anisotropy depolarization [file 42004_2026_1994_MOESM2_ESM.pdf]

Supporting Information for

Recombinant protein platform for high-throughput investigation of peptide-liposome interactions via fluorescence anisotropy depolarization

Antonis Margaritakis[1], Meirui Qian[2], David H. Johnson[3], Wade F. Zeno[3], Tobias S. Ulmer[2] and Peter J. Chung[1,4,5*]

[1]Department of Physics and Astronomy, University of Southern California, Los Angeles, California, USA. [2]Department of Physiology and Neuroscience, Zilkha Neurogenetic Institute, Keck School of Medicine, University of Southern California, Los Angeles, California, USA. [3]Mork Family Department of Chemical Engineering and Materials Science, University of Southern California, Los Angeles, California, USA. [4]Department of Chemistry, University of Southern California, Los Angeles, California, USA. [5]Alfred E. Mann Department of Biomedical Engineering, University of Southern California, Los Angeles, California, USA.

*Corresponding Author

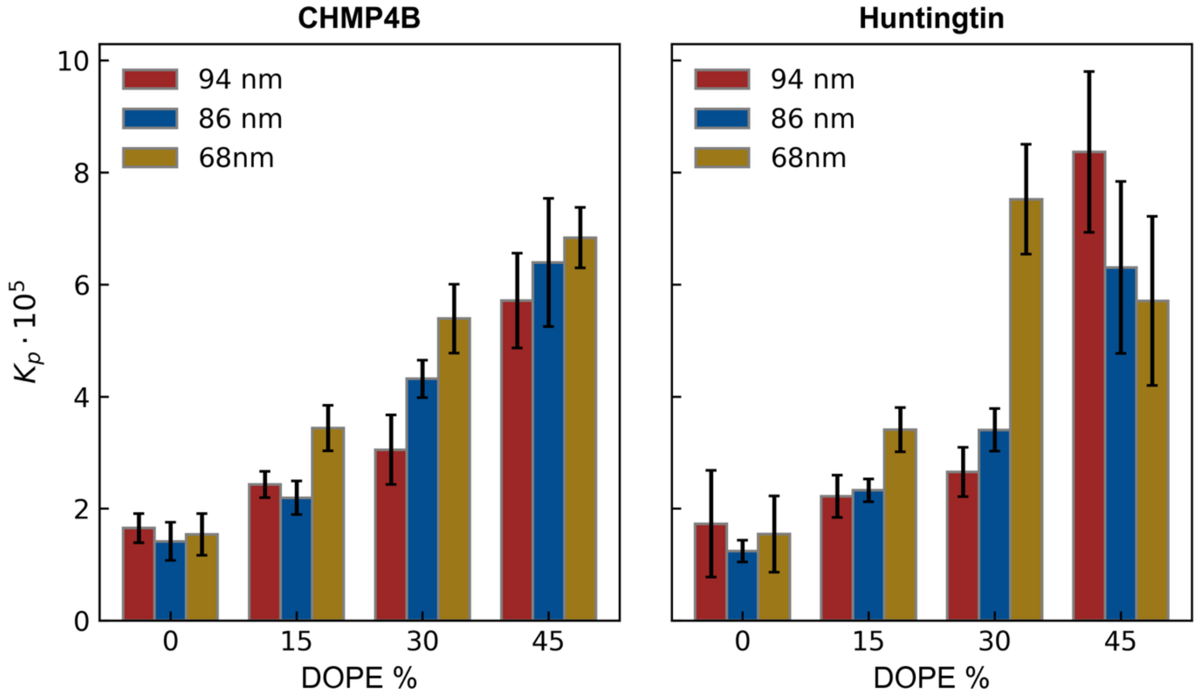

Figure S1 | Fitted partition coefficient values from fluorescence anisotropy data of Figure 2. Error bars represent standard errors of each fit. Vesicles composition in each case is DOPC/DOPS/DOPE : (70-X)/30/X where X is the percentage shown on the x-axis. Legend shows measured average vesicle diameter.

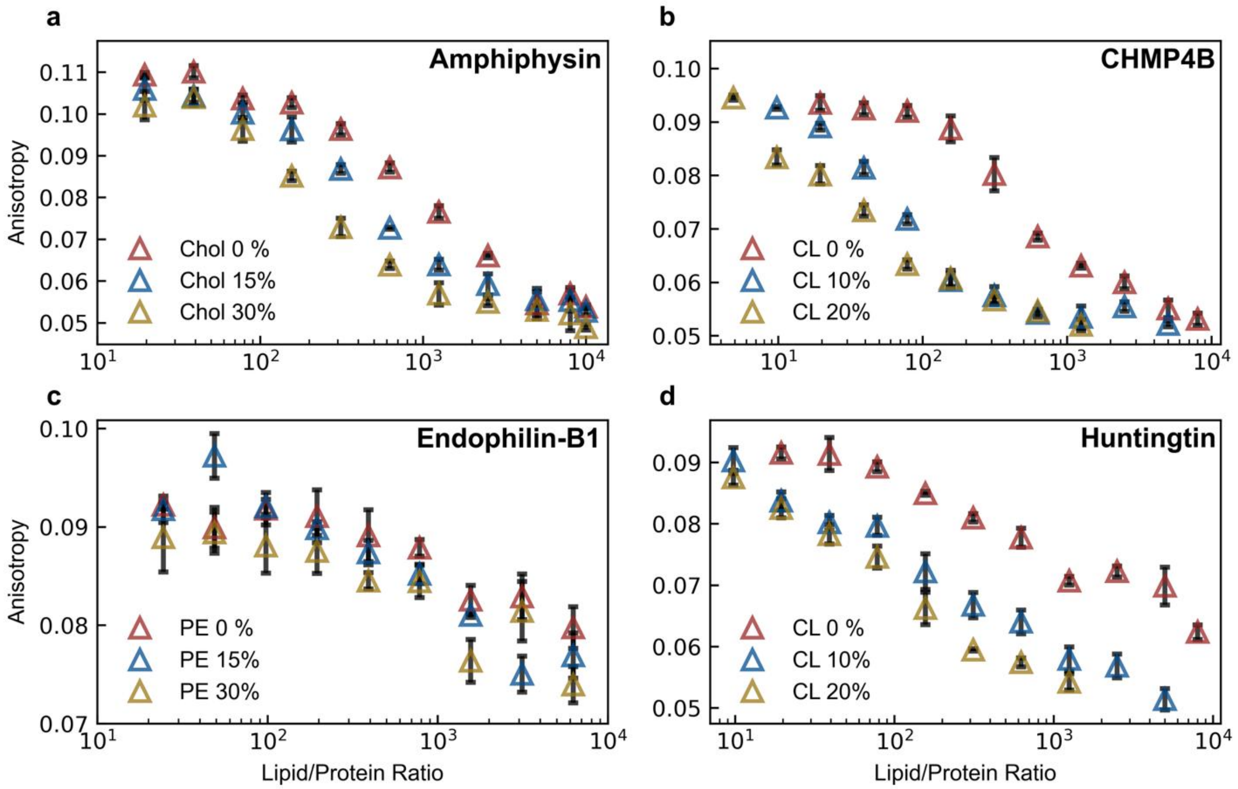

Figure S2 | Fluorescence anisotropy measurements of the 4 peptide constructs shown in Figure 3. Each platform was tested against a different array of liposome compositions as described in Fig 3. Data points show mean of 4 replicate wells for each lipid/protein ratio, with standard deviations plotted (black error bars).

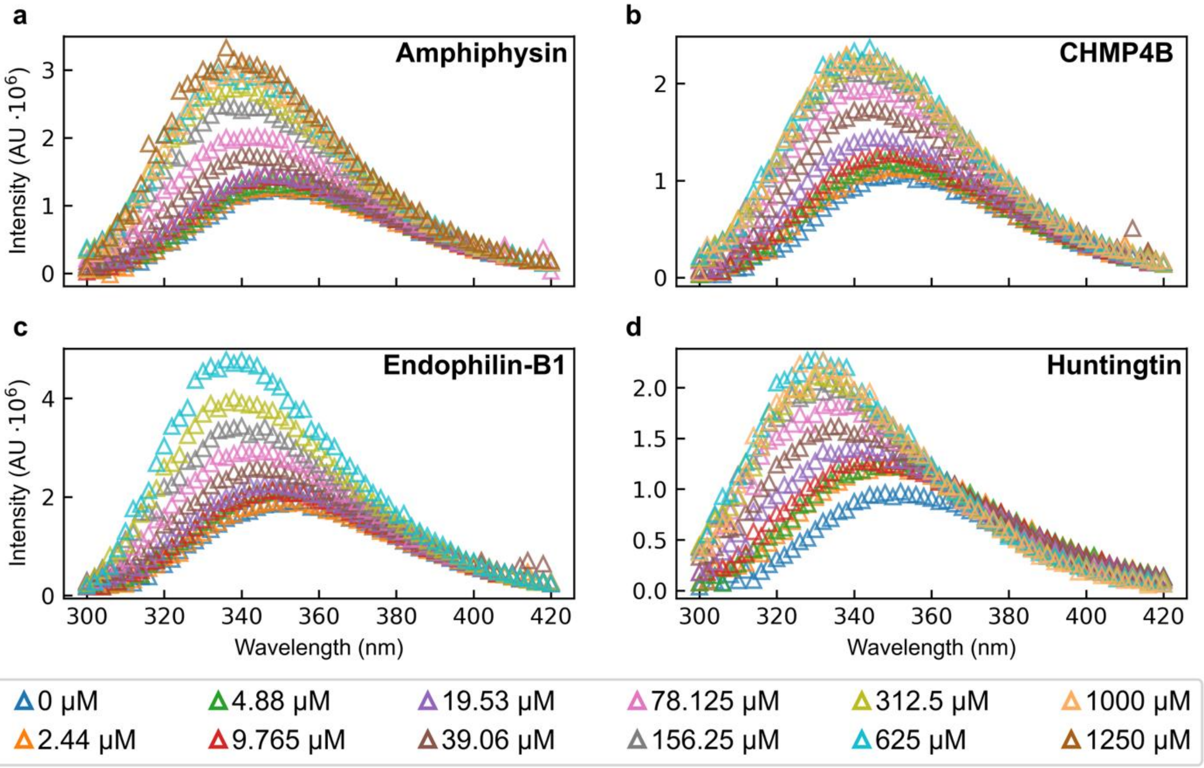

Figure S3 | Representative tryptophan fluorescence measurement spectra from data shown in Figure 3. Tryptophan fluorescence spectra of our protein platforms including inducible the amphipathic helices of **(a)** Amphiphysin (AA: 1-25, F9W, 125 nM), **(b)** Endophilin-B1 (AA: 1-33, F18W, 100 nM), **(c)** Huntingtin (AA: 1-17, F11W, 125 nM) and **(d)** CHMP4B (AA: 1-19, F8W, 125 nM) measured against varying concentrations (legend) of DOPC/DOPS:70/30 liposomes with size distribution averages of ~70 nm.

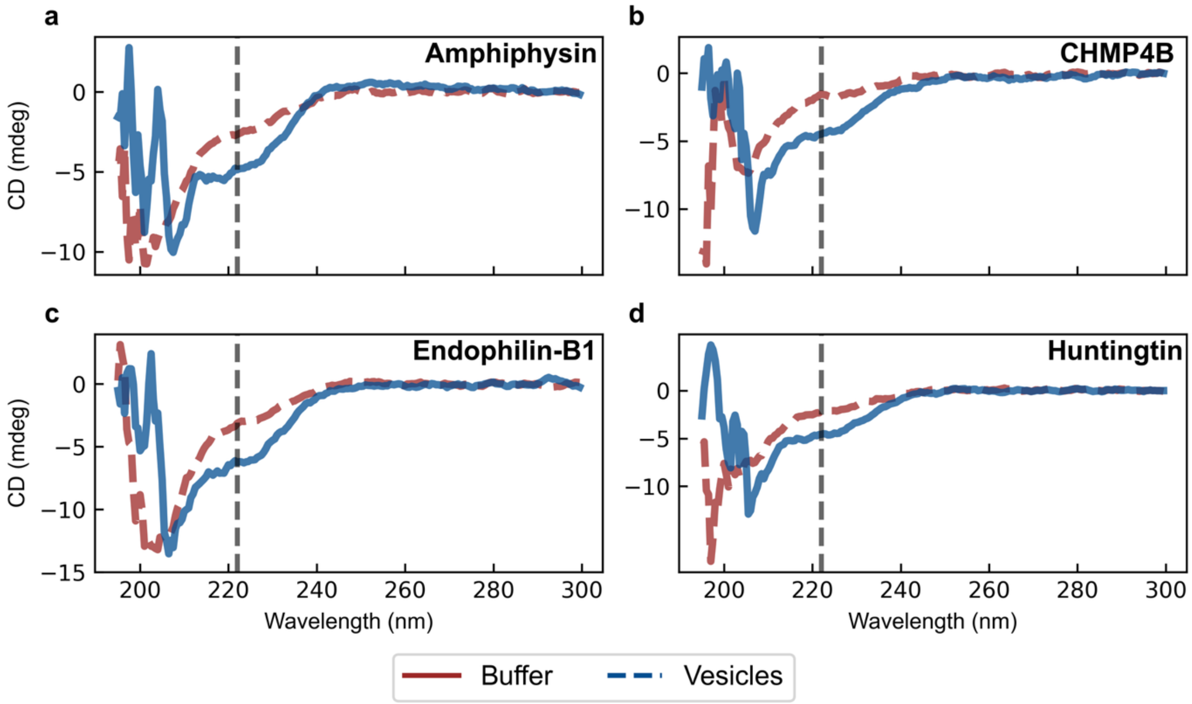

Figure S4 | Peptides transition into amphipathic helices upon interacting with liposomes. Circular dichroism spectra of our protein platforms (8 μM) including the inducible amphipathic helices of **(a)** Amphiphysin (AA: 1-25, F9W), **(b)** CHMP4B (AA: 1-19, F8W), **(c)** Endophilin-B1 AA: 1-33, F18W) and **(d)** Huntingtin (AA: 1-17, F11W) measured against 4 mM of DOPC/DOPS:70/30 vesicles with size distribution averages of ~68 nm. Characteristic decrease in CD signal at 222 nm (black dashed line) indicates the formation of an amphipathic helix.

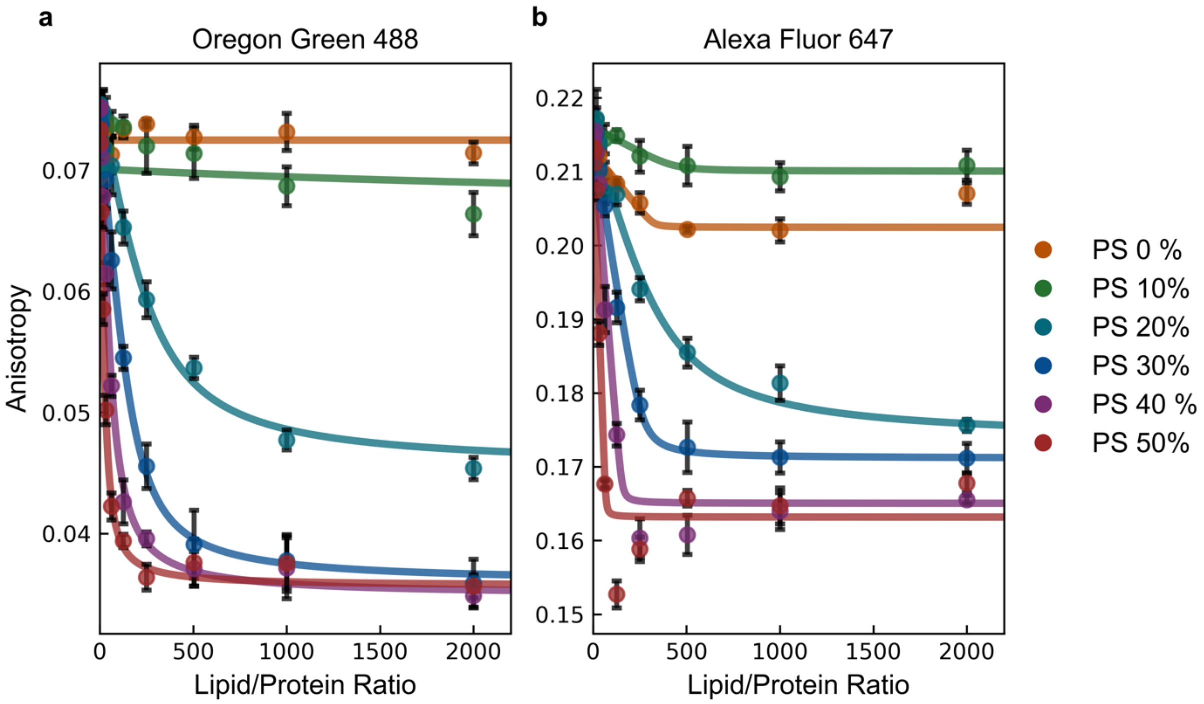

Figure S5 | The decrease in fluorescence anisotropy is independent of fluorophore identity. Fluorescence anisotropy measurements of differently fluorescently-tagged hexahistidine model platforms (@ 250 nM concentration) binding to similarly composed nickel-chelating lipid containing vesicles with varying amounts of DOPS membrane content (DOPC/DOPS/DGS-NTA(Ni) = 90-X/X/10). Data shown are averages of n=4 replicate wells with standard deviations plotted (black error bars).

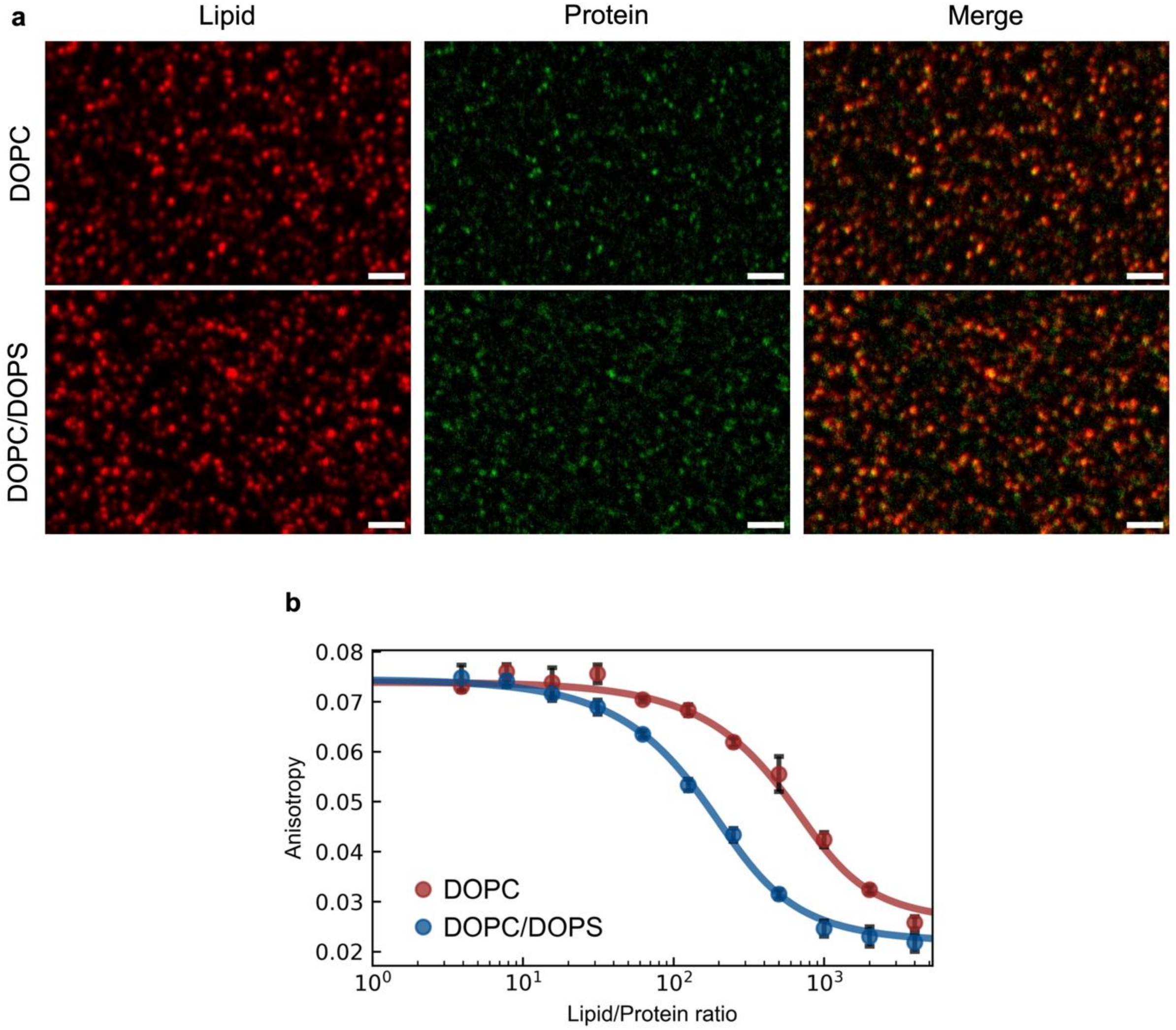

Figure S6 | Confocal microscopy images confirm binding of hexahistidine model platform to liposomes. **(a)** Confocal microscopy images from tethered vesicle assay reveal binding of the fluorescently-tagged hexahistidine model platform to vesicles compositions of DOPC/DGS-NTA(Ni)/Biotinyl/DPPE-Atto647 = 87.5/10/2/0.5 vesicles and DOPC/DOPS/DGS-NTA(Ni)/Biotinyl/DPPE-Atto647 = 47.5/40/10/2/0.5. A high pass filter was applied on the image of the protein channel. **(b)** Fluorescence anisotropy response of the fluorescently-tagged hexahistidine model platform with the same vesicles as above. A decrease in anisotropy is now present even with low charged vesicles. Data shown are averages from n=3 replicate wells with standard deviations plotted (black error bars).

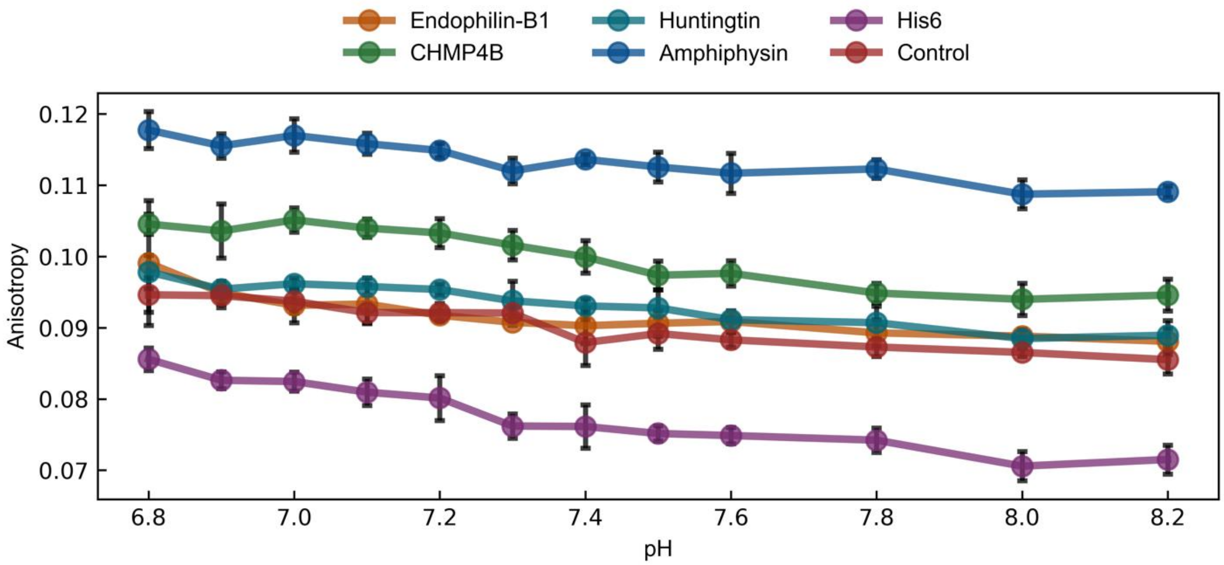

Figure S7 | Fluorescence anisotropy response of inducible amphipathic helix peptide and control platforms in varying pH. Fluorescence anisotropy measurements against a pH gradient (20 mM HEPES) were taken for all of all 6 recombinant protein platforms (250 nM) used in this paper. “Control: referes to the fluorescently-labeled recombinant platform with no peptide on its N-terminus as in Figure S2. Data points show mean of n=4 replicate wells for each lipid/protein ratio, with standard deviations plotted (black error bars).

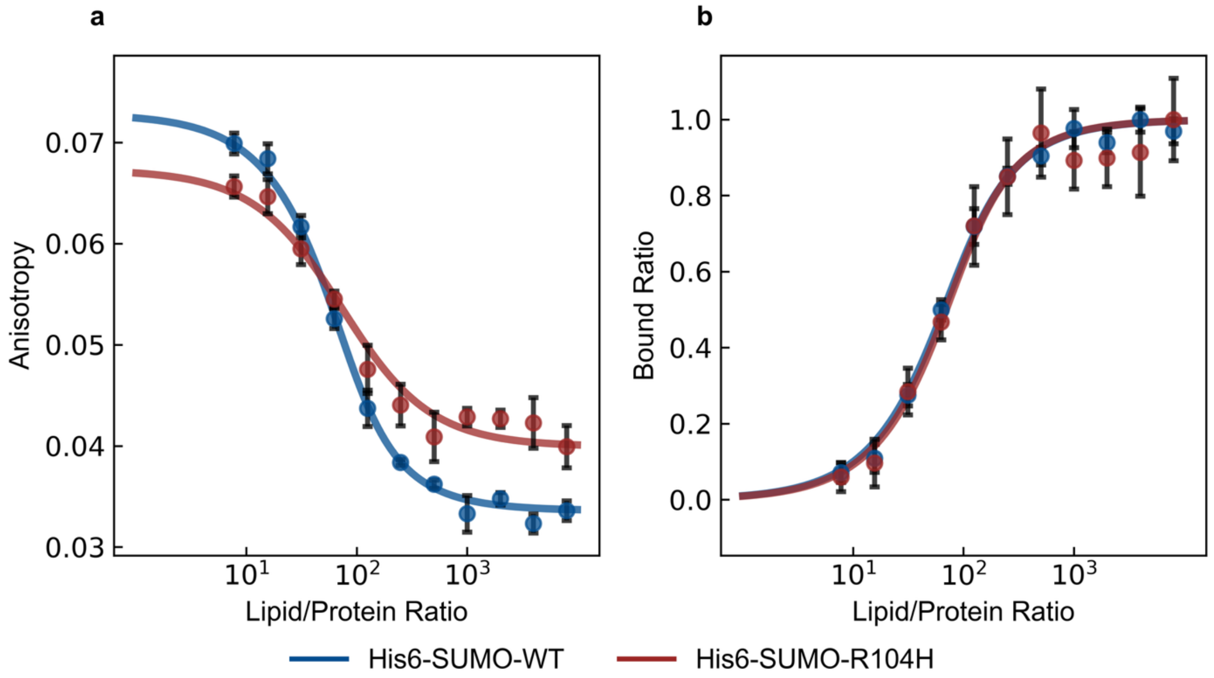

Figure S8 | Binding response of the fluorescently-tagged hexaxistidine model platform and the mutated R104H model platform to nickel-chelating containing lipid vesicles. **(a)** Raw fluorescence anisotropy data from mutated model R104H hexahistidine platform (250 nM) binding to vesicles (with a composition of DOPC/DOPS/DGS-NTA(Ni) = 50/40/10 and an average diameter of ~95 nm) and **(b)** transformed data into bound ratio curves as described in Methods. Data shown are averages from n=4 replicate wells with standard deviations plotted (black error bars).

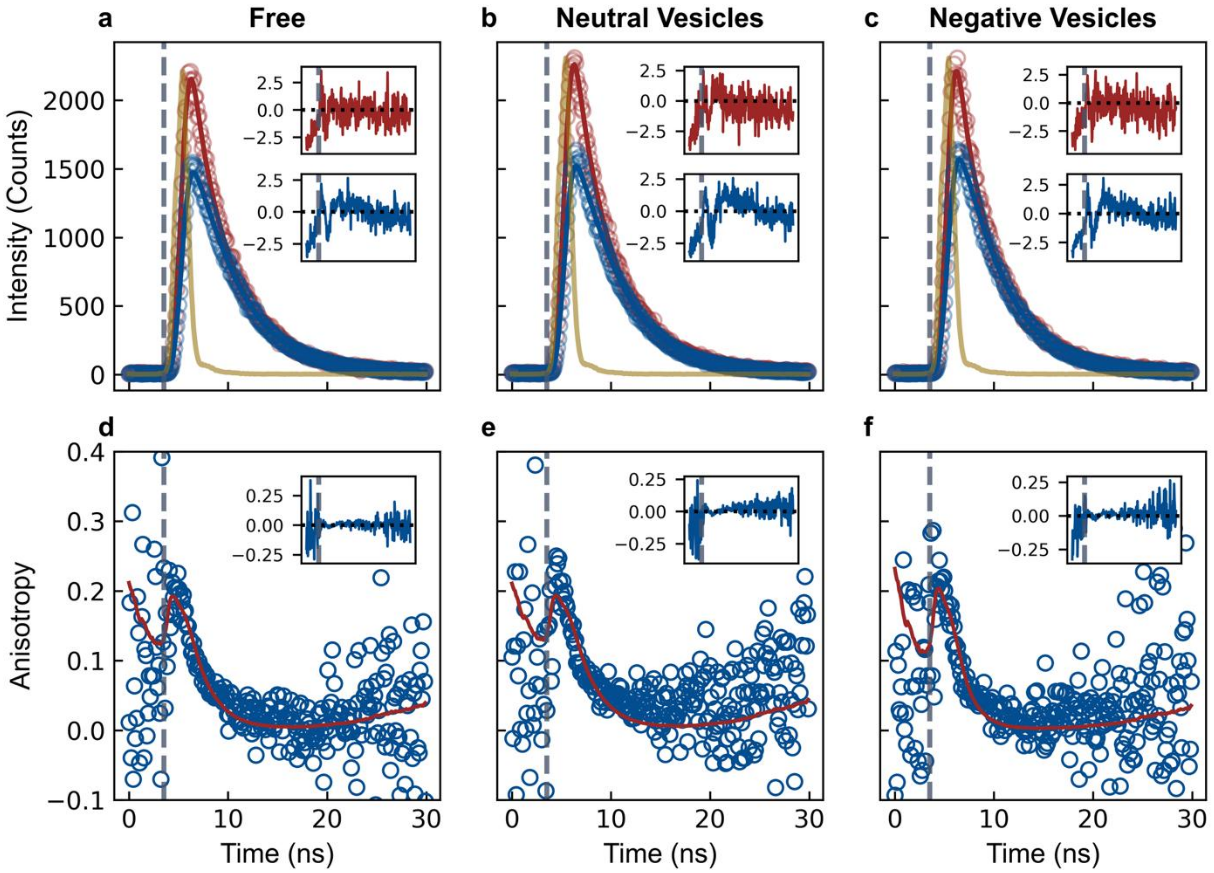

Figure S9 | Anisotropy decay data and fits with single rotational correlation lifetime model. Parallel (blue) and perpendicular (red) intensity decays and corresponding fits of the fluorescently-tagged hexahistidine model platform (25 nM) **(a)** free in solution **(b)** bound to neutral vesicles (DOPC/DGS-NTA(Ni) = 90/10, 25 µM) and **(c)** bound to negatively charged vesicles (DOPC/DOPS/DGS-NTA(Ni) = 50/40/10, 25 µM). **(d-f)** Clalculated anisotropy decays and fits from (a-c) respectively. Insets show residuals of each fit. Vertical dashed grey line indicated start of pulse. Yellow curve indicates the instrument response function.

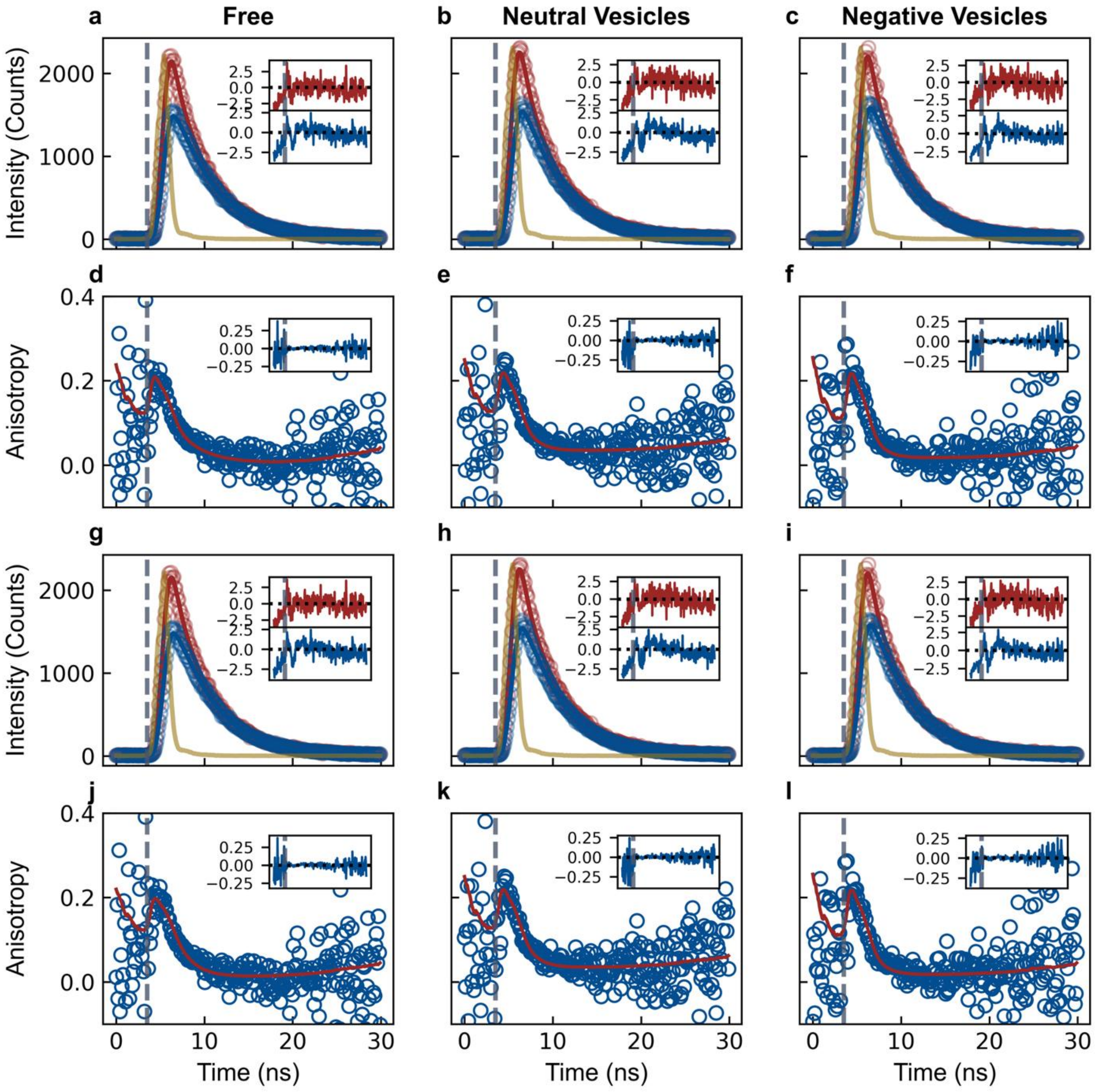

Figure S10 | Anisotropy decay data of fluorescently-tagged hexahistidine model platfrom fitted with 2 different models. Anisotropy decay data from Figure S11 fitted with 2 different models shown in 3 columns (**Free**: protein free in solution, **Neutral Vesices**: protein bound to vesicles comprised of DOPC/DGS-NTA(Ni) = 90/10, **Negative Vesicles**: bound to negatively charged vesicles (DOPC/DOPS/DGS-NTA(Ni) = 50/40/10). **(a-c)** Parallel (blue) and perpendicular (red) intensity decays and corresponding fits using the two-state hindered anisotropy decay model. **(d-f)** Clalculated anisotropy decays and fits from (a-c) respectively. **(g-i)** Parallel (blue) and perpendicular (red) intensity decays and corresponding fits using reduced two-state model (hindered rotational diffusion) model. **(j-l)** Clalculated anisotropy decays and fits from (g-i) respectively. Insets show residuals of each fit. Vertical dashed grey line indicated start of pulse. Yellow curve indicates the instrument response function. Fitted values are shown in Table S1.

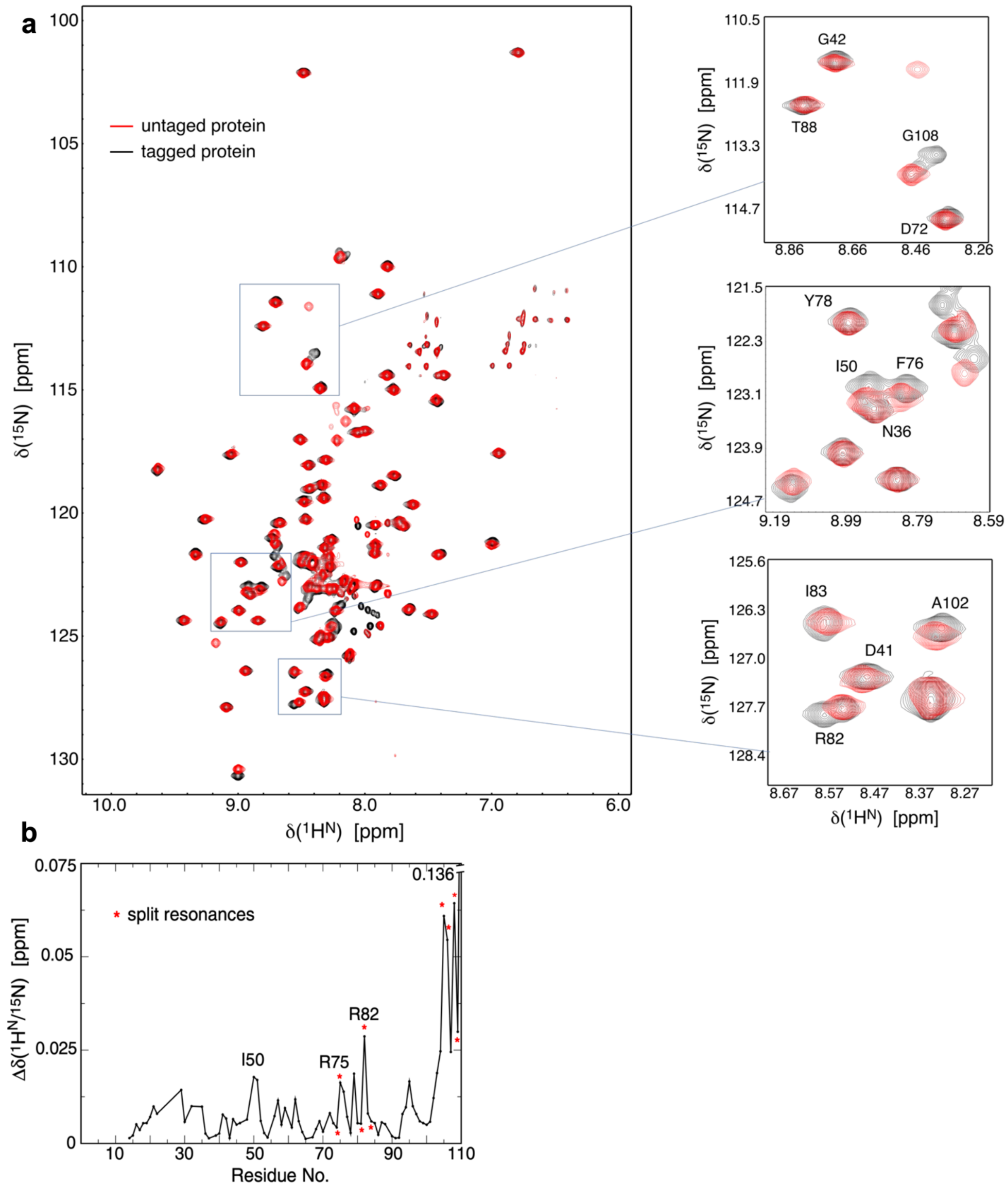

Figure S11 | Comparison of $^{1}H^{N}$-^{15}N correlation spectra of fluorescently-tagged and untagged hexahistidine model platform. (a) For C-terminal residues shifted peaks and resonance splitting were prevalent. Nonetheless, this pattern also occurred within the SUMO domain as indicated. **(b)** Chemical shift difference between Oregon Green 488 tagged and untagged hexahistidine model platform. Some residues

exhibited doubled resonances. For such split peaks, the most intense signal was considered to represent the fluorophore-labeled state.

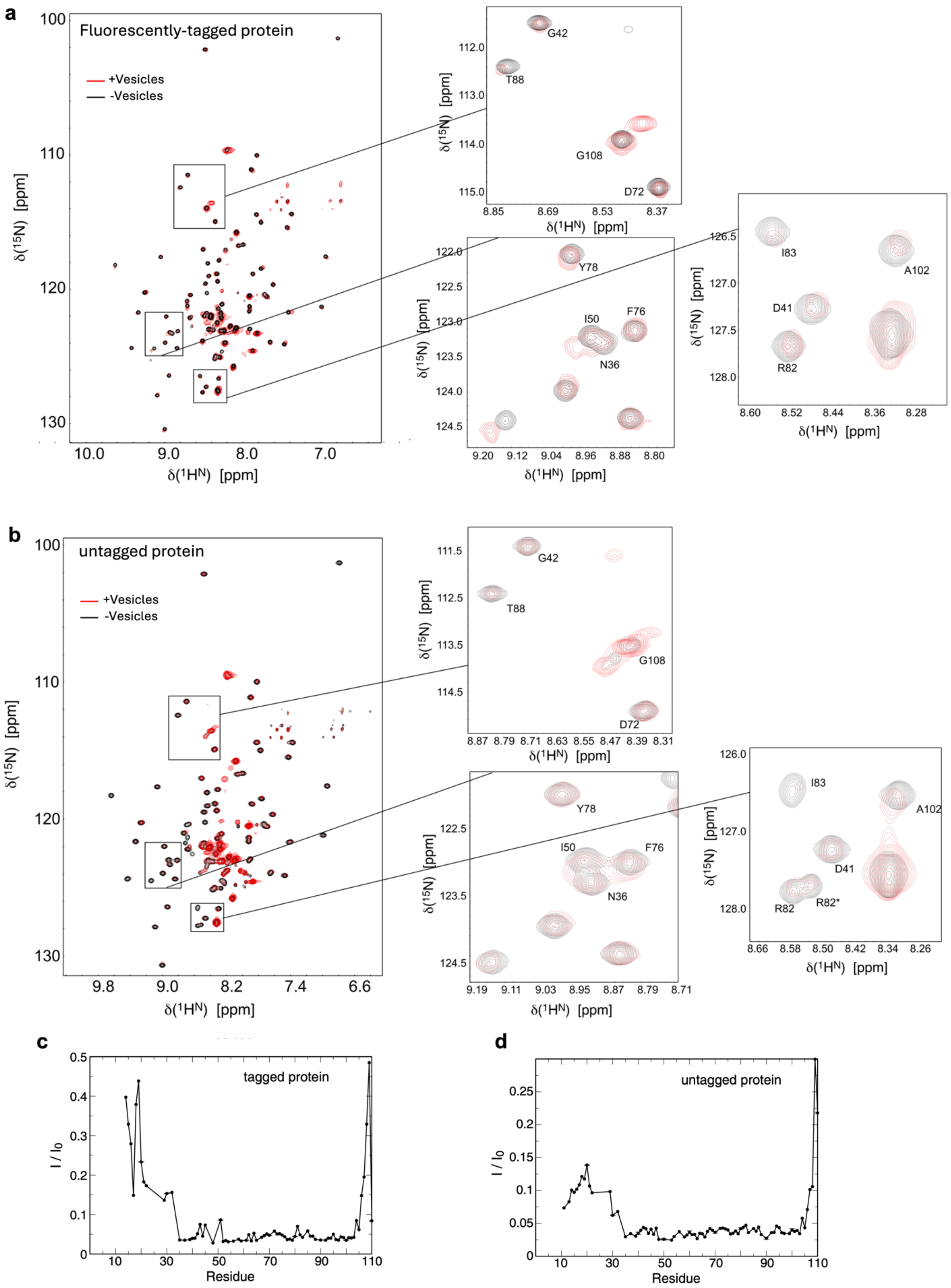

a
Fluorescently-tagged protein
+Vesicles
-Vesicles
δ(15N) [ppm]
δ(1HN) [ppm]
G42
T88
G108
D72
Y78
I50
F76
N36
I83
A102
D41
R82
b
untagged protein
+Vesicles
-Vesicles
δ(15N) [ppm]
δ(1HN) [ppm]
G42
T88
G108
D72
Y78
I50
F76
N36
I83
A102
D41
R82
R82*
c
tagged protein
I / I0
Residue
d
untagged protein
I / I0
Residue

Figure S12 | Comparison of $^{1}H^{N}$-^{15}N correlation spectra of free and vesicle-bound hexahistidine model platform. (a) Correlation spectra of fluorescently-tagged and **(b)** untagged hexahistidine model platform when free in solution vs when partially bound to vesicles comprised of DOPC/DOPS/DGS-NTA(Ni) = 50/40/10. In the presence of vesicles, spectral positions of the protein platform remained close to the vesicle-free state with reduced signal intensities, indicating predominantly slow exchange kinetics between free and vesicle-bound protein. Because of the large overall particles size, resonances of vesicle-bound protein are unobservable. **(c, d)** Residual $^{1}H^{N}$-^{15}N cross-peak intensities of fluorescently-tagged and untagged hexahistidine model platform in the presence of vesicles. For both platforms, the N-terminal region (Met1-Glu32), the SUMO domain, and the C-terminal region (Gln106-Cys110) exhibit different degrees of signal reductions, illustrating their flexible coupling.

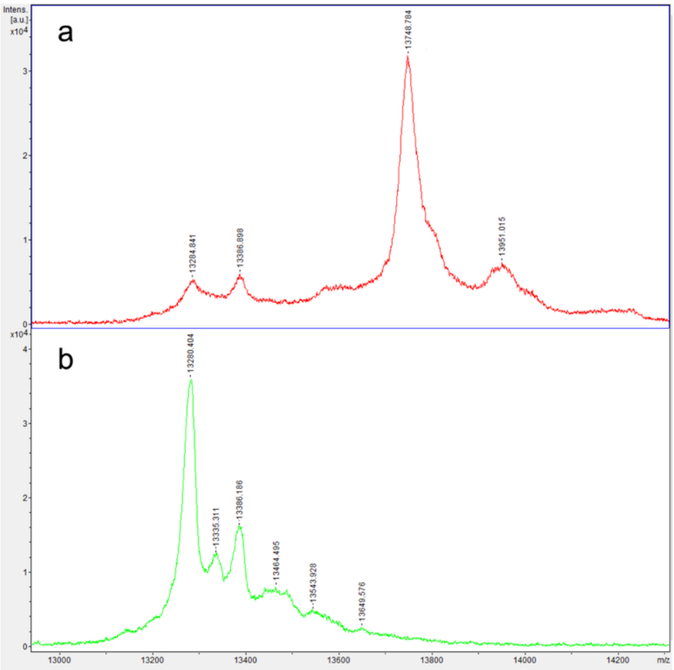

Figure S13 MALDI-TOFF mass spectrometry of the (a) fluorescently labeled and (b) unlabeled $^{1}H^{N}$-^{15}N hexahistidine model platform. Quantitative analysis based on peak areas indicates that approximately 78.6% of the protein sample is fluorescently labeled, 8% is unlabeled, and the remaining 13.5% consists of alternative protein forms.

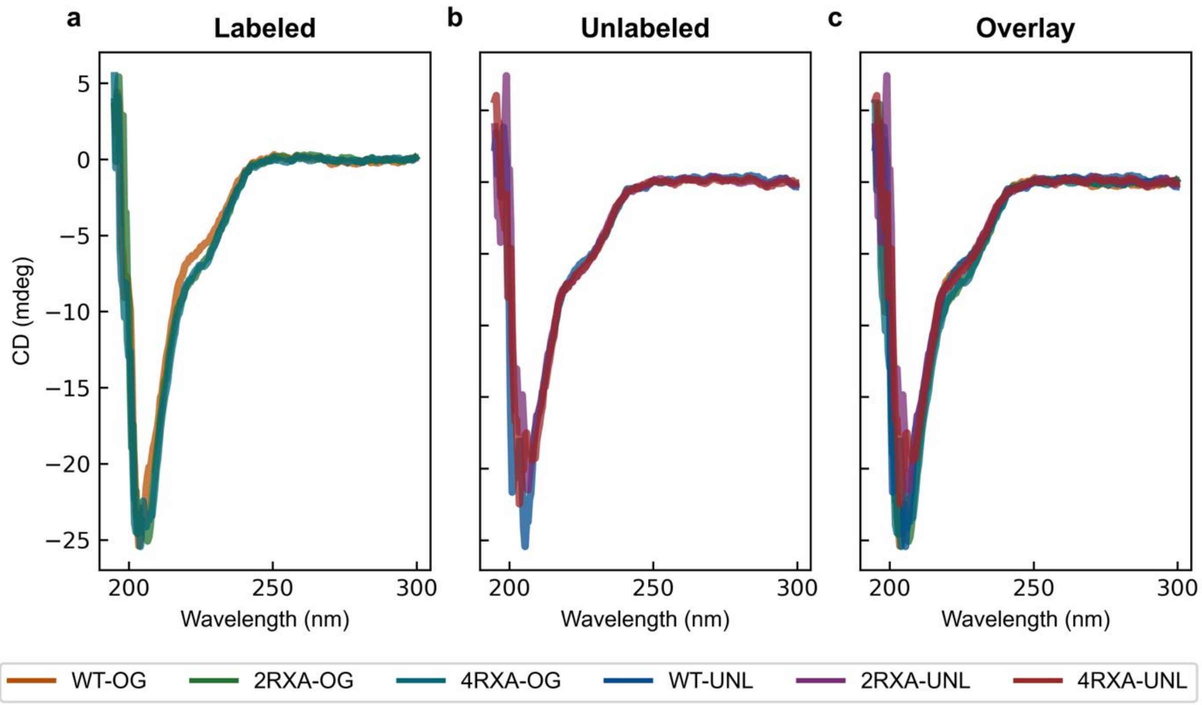

Figure S14. Arginine to Alanine mutations do not alter structure of hexahistidine model construct. Circular dichroism measurements for (a) OG488 fluorescently labeled His_6-SUMO constructs, (b) unlabeled constructs and (c) overlay of (a) and (b). The three constructs tested are the wild type His_6-SUMO platform (WT), the His_6-SUMO-R75A-R82A (2RXA) and the His_6-SUMO-R57A-R75A-R82A-R104A (2RXA) mutants. Protein concentration was 20 mM in buffer(20 mM Hepes at pH 7).

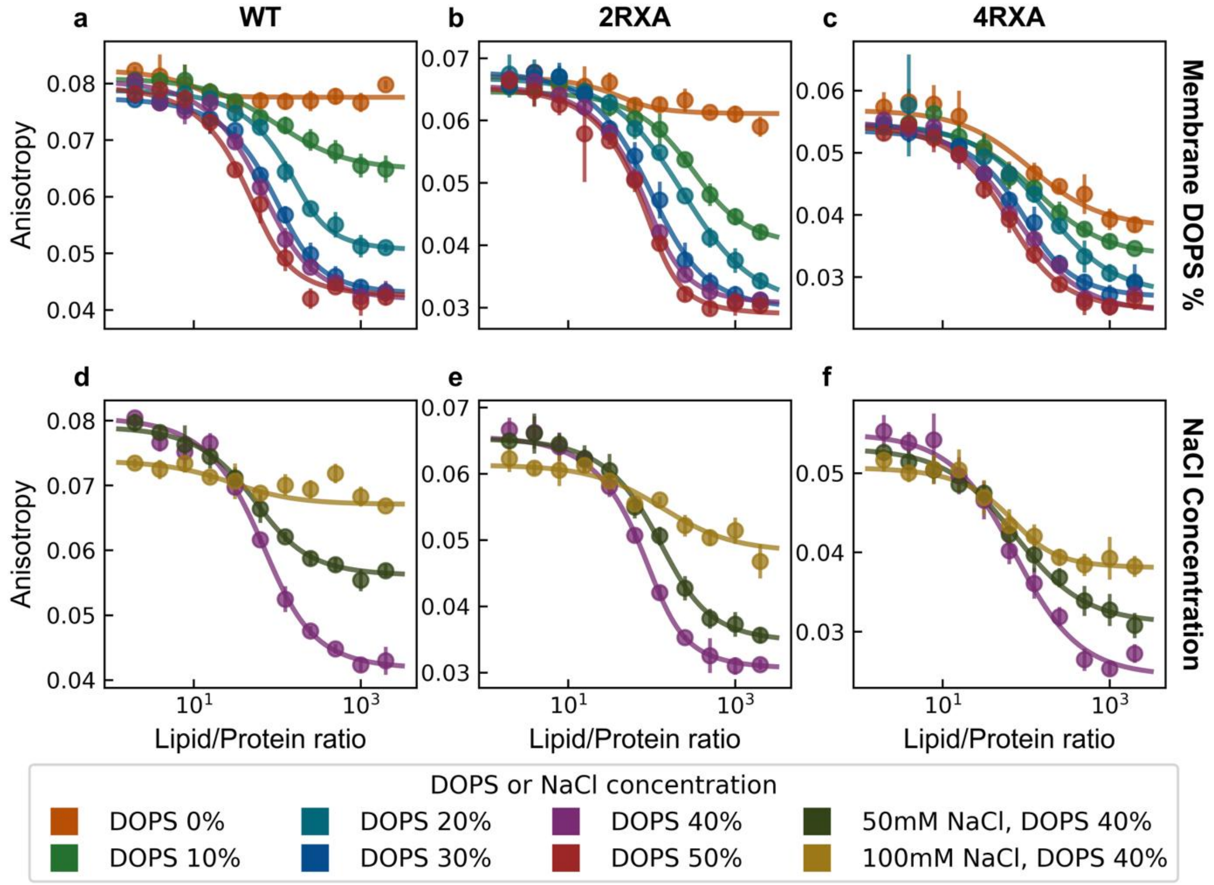

Figure S15 Fluoresce anisotropy decrease viewing window of mutated His$_6$-SUMO-fluorophore constructs greatly increases. The fluorescence anisotropy response was measured for 3 version of the hexahistidine model platform binding to nickel-chelating lipid containing vesicles against different conditions: **(a-c)** membrane charge (with compositions of DOPC/DOPS/DGS-NTA(Ni) = (90-X)/X/10 with X shown in the legend) and **(d-f)** added NaCl concentration (with lipid vesicle compositions of DOPC/DOPS/DGS-NTA(Ni) = 50/40/10). Data points are the average of n=4 replicate wells for each lipid/protein ratio with standard deviations plotted (error bars). Solid lines represent fits to a depletion model as described in methods. Vesicles were extruded to a final diameter of 90-100 nm. Added NaCl concentration is 0 where not listed. Each column represents measurements for a single His$_6$-SUMO construct with or without arginine-to-alanine mutations.

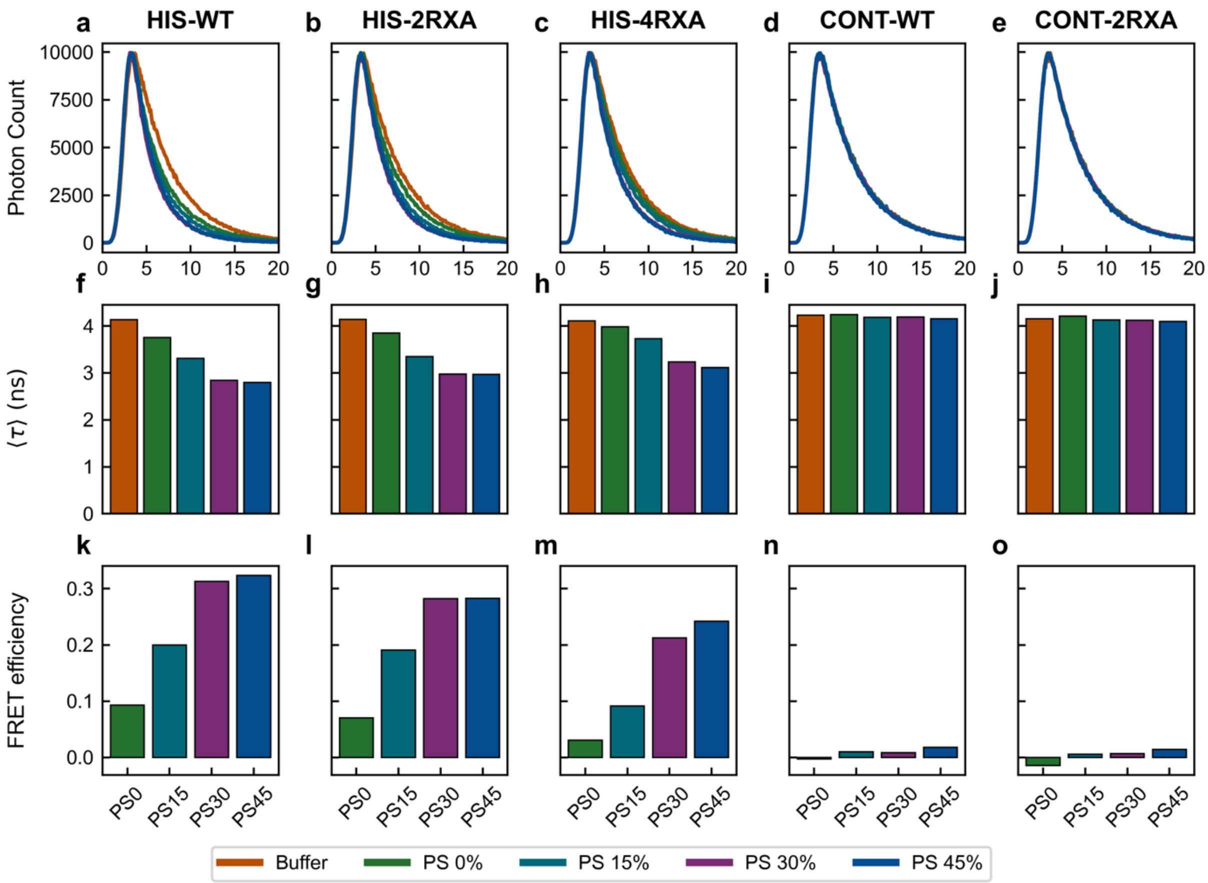

Figure S16 | Fluorescence lifetime decay of OG488-labeled protein constructs bound to lipid vesicles as a function of membrane phosphatidylserine (PS) concentration. (a–e) Donor OG488 fluorescence lifetime decays under conditions permitting FRET with the liposome headgroup–conjugated acceptor DOPE–Liss-Rhodamine-B, for proteins free in solution and bound to vesicles. Vesicles were composed of DOPC/DOPS/DGS-NTA(Ni))/DOPE-Liss-Rhodamine-B = (88–X)/X/10/2, with X indicated in the legend. Protein and vesicle concentrations were 10 nM and 25 µM, respectively. “HIS” constructs contain an N-terminal hexahistidine tag and varying arginine-to-alanine mutations within the SUMO domain, whereas “CONT” constructs lack the hexahistidine tag. All CONT constructs exhibit single, unaltered fluorescence decays under all conditions, indicating no interaction with vesicles. **(f–j)** Mean fluorescence lifetimes (see Methods) for protein constructs under varying membrane PS concentrations and in solution. Increasing PS content in the membrane results in decreased mean fluorescence lifetimes for all HIS constructs. **(k–o)** FRET efficiencies calculated from the corresponding mean fluorescence lifetimes. HIS constructs exhibit measurable FRET in the presence of vesicles at all PS concentrations, with FRET efficiency increasing with membrane DOPS content. Mutant constructs display progressively reduced FRET responses at constant PS concentrations. Each column corresponds to data from a single protein construct.

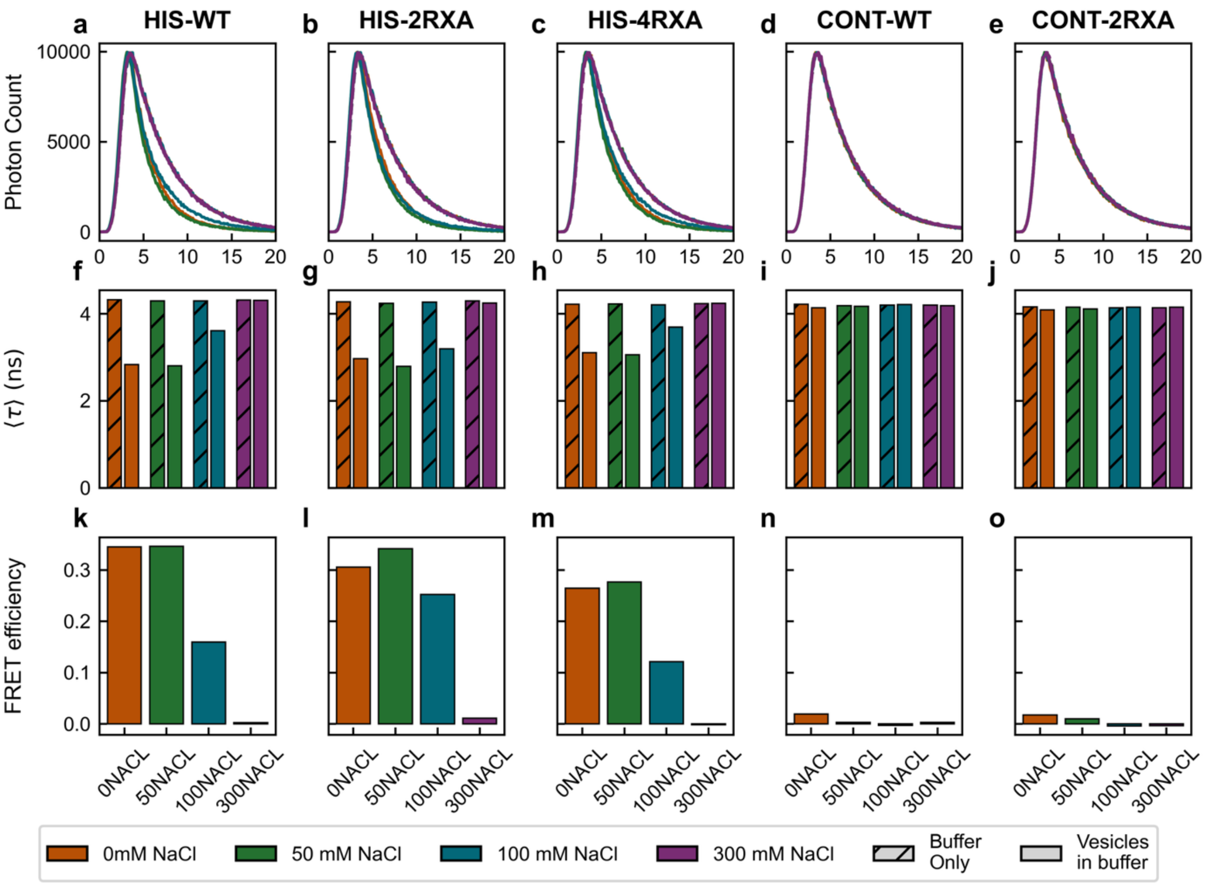

Figure S17 | Fluorescence lifetime decay of OG488-labeled protein constructs bound to lipid vesicles as a function of NaCl concentration. (a–e) Donor OG488 fluorescence lifetime decays under conditions permitting FRET with the liposome headgroup–conjugated acceptor DOPE–Liss-Rhodamine-B, for proteins free in solution and bound to vesicles. Vesicles composition was DOPC/DOPS/DGS-NTA(Ni))/DOPE-Liss-Rhodamine-B = 48/40/10/2 everywhere. Protein and vesicle concentrations were 10 nM and 25 µM. “HIS” constructs contain an N-terminal hexahistidine tag and varying arginine-to-alanine mutations within the SUMO domain, whereas “CONT” constructs lack the hexahistidine tag. All CONT constructs exhibit single, unaltered fluorescence decays under all conditions, indicating no interaction with vesicles. Decays in buffer conditions are represented with dashed lines, whereas solid lines are for vesicle containing samples. **(f–j)** Mean fluorescence lifetimes (see Methods) for protein constructs under varying NaCl concentrations free in solution and with vesicles. Increasing NaCl content results in decreased mean fluorescence lifetimes for all HIS constructs. **(k–o)** FRET efficiencies calculated from the corresponding mean fluorescence lifetimes. “HIS” constructs exhibit measurable FRET in the presence of vesicles at all NaCl concentrations, except at 300mM, with FRET efficiency decreasing with increased NaCl. Mutant constructs display progressively reduced FRET responses at constant NaCl concentrations. Each column corresponds to data from a single protein construct.

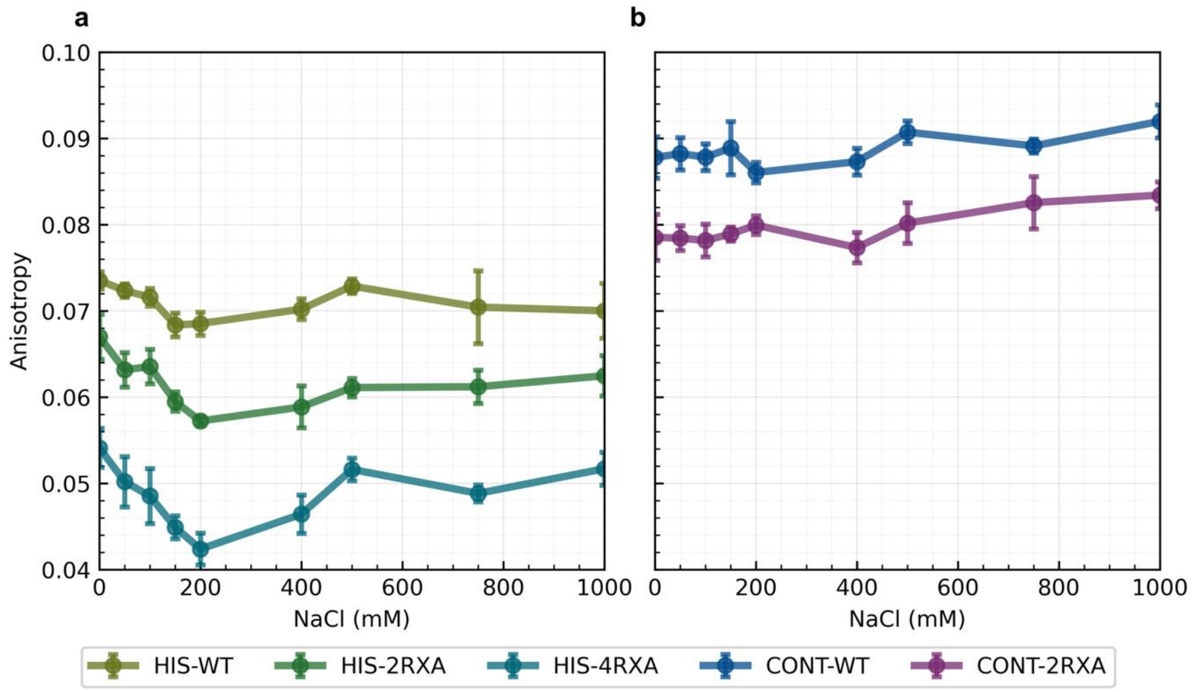

Figure S18 | Steady state fluorescence anisotropy decreases for mutant constructs lacking the limited arginine patch in the SUMO domain, indicating enhanced local mobility for the fluorophore. Free protein measurements were taken on a plate reader for 100 nM of protein in buffer containing 20 mM HEPES, pH 7 with increasing environmental NaCl concentrations. CONT refers to SUMO constructs without a N-terminal His_6 tag. Data shown are averages of n=4 replicate wells with standard deviations plotted (error bars).

Table S1 | Fitted parameter values from anisotropy model fits. 1r: single rotational correlation lfietime model, 2r: two-state hindered anisotropy decay model, 2r-H-red: reduced two-state model (hindered rotational diffusion) model

	**I_0 (AU)**	**τ (ns)**	**r_0**	**α**	**θ_F (ns)**	**θ_P (ns)**	**r_{inf}**	**θ_{cone} (°)**
**1r Free**	7290±30	4.25±0.01	0.212±0.007	-	-	1.9±0.1	-	-
**1r Bound Low charge**	7610±40	4.18±0.01	0.211±0.007	-	-	2.2±1.1	-	-
**1r Bound High charge**	7660±30	4.17±0.01	0.23±0.01	-	-	1.30±0.09	-	-
**2r Free**	7290±30	4.24±0.01	0.24±0.02	0.6±0.3	1.1±0.7	3±1	-	40±10
**2r Bound Low charge**	7630±30	4.16±0.01	0.25±0.01	0.87±0.03	1.1±0.1	~∞	-	61±3
**2r-H Bound High charge**	7670±30	4.15±0.01	0.25±0.01	0.94±0.03	0.9±0.1	~∞	-	69±4
**2r-H-red Bound Low charge**	7630±30	4.16±0.01	0.25±0.01	-	1.1±0.1	-	0.033±0.004	61±2
**2r-H-red Bound High charge**	7670±30	4.15±0.01	0.25±0.01	-	0.93±0.09	-	0.016±0.004	69±2

Supplementary Notes 1

Hindered Rotational Diffusion in Membranes

In order to effectively evaluate the fitted fluorescence anisotropy decay data and produce a more comprehensive geometric interpretation of the phenomena detected, we employ an interpretation of the hindered rotation describing the molecule as rod-like in an infinite square-well potential so that its rotation is unhindered until a certain angle θ_C is reached[1–3]. In this model the limiting and time zero anisotropy are related to the cone angle:

$$\frac{r_\infty}{r_0} = \left(\frac{1}{2} cos\,\theta_C (1 + cos\,\theta_C)\right)^2$$

with completely unhindered motion for θ_C= 90°. This approach can be expanded to the dual rotational diffusion with hindered segmental mobility model by considering the relationship between a and r_∞: $r_\infty = r_0(1 - a)$ and hence extract an estimated angle for the cone of motion for the local hindered diffusion of the fluorophore. For the free state we calculate a θ_C≈40±10° whereas for the bound state on neutral and negative vesicles it is θ_C≈61±2° and θ_C≈69±2° respectively.

Supplementary References

1. Kinosita, K., Kawato, S. & Ikegami, A. A theory of fluorescence polarization decay in membranes. *Biophys. J.* **20**, 289–305 (1977).

2. Kinosita, K., Kawato, S. & Ikegami, A. Dynamic structure of biological and model membranes: analysis by optical anisotropy decay measurement. *Adv. Biophys.* **17**, 147–203 (1984).

3. *Principles of Fluorescence Spectroscopy*. (Springer US, Boston, MA, 2006). doi:10.1007/978-0-387-46312-4.
