# Supplementary material for: Recombinant protein platform for high-throughput investigation of peptide-liposome interactions via fluorescence anisotropy depolarization
Source: Commun Chem. 2026 Apr 2;9:165. doi: 10.1038/s42004-026-01994-9 (PMC13106830; doi:10.1038/s42004-026-01994-9)
Supplement: Supplementary file 3 — Description of Additional Supplementary Files [file 42004_2026_1994_MOESM3_ESM.pdf]

Description of Additional Supplementary Files:

File: Supplementary Data

Description: All data used to derive plots for this paper
